# Erasure and reestablishment of random allelic expression imbalance after epigenetic reprogramming

**DOI:** 10.1261/rna.058347.116

**Published:** 2016-10

**Authors:** Aaron Richard Jeffries, Dafe Aghogho Uwanogho, Graham Cocks, Leo William Perfect, Emma Dempster, Jonathan Mill, Jack Price

**Affiliations:** 1 University of Exeter Medical School, University of Exeter, Exeter EX2 5DW, United Kingdom; 2 Institute of Psychiatry, Psychology and Neuroscience, King's College London, London SE5 8AF, United Kingdom

**Keywords:** RNA, allelic expression, DNA methylation, stem cells, monoallelic, iPSC

## Abstract

Clonal level random allelic expression imbalance and random monoallelic expression provides cellular heterogeneity within tissues by modulating allelic dosage. Although such expression patterns have been observed in multiple cell types, little is known about when in development these stochastic allelic choices are made. We examine allelic expression patterns in human neural progenitor cells before and after epigenetic reprogramming to induced pluripotency, observing that loci previously characterized by random allelic expression imbalance (0.63% of expressed genes) are generally reset to a biallelic state in induced pluripotent stem cells (iPSCs). We subsequently neuralized the iPSCs and profiled isolated clonal neural stem cells, observing that significant random allelic expression imbalance is reestablished at 0.65% of expressed genes, including novel loci not found to show allelic expression imbalance in the original parental neural progenitor cells. Allelic expression imbalance was associated with altered DNA methylation across promoter regulatory regions, with clones characterized by skewed allelic expression being hypermethylated compared to their biallelic sister clones. Our results suggest that random allelic expression imbalance is established during lineage commitment and is associated with increased DNA methylation at the gene promoter.

## INTRODUCTION

Although autosomal genes are predominantly expressed from both the maternal and paternal alleles, examples of allelic imbalance or monoallelic expression have been described. Genotype-associated allelic imbalances, regulated by expression quantitative trait loci (eQTLs) in *cis*, are acknowledged to be a widespread source of phenotypic variation in humans (Ge et al. 2009) and mouse (Crowley et al. 2015). Monoallelic expression can also be epigenetically controlled in a parent-of-origin specific manner, as exemplified by genomic imprinting (Reik and Walter 2001), a relatively rare phenomenon affecting approximately 100 genes in human and 120–180 genes in mouse (Babak et al. 2015; Baran et al. 2015; Crowley et al. 2015). Monoallelic gene expression can also be random, whereby a cell stochastically expresses a single allele. This phenomenon has been well described in female mammalian cells, which transcriptionally inactivate the majority of genes on one randomly chosen X chromosome (Lyon 1986; Carrel and Willard 2005). X-chromosome inactivation occurs during the morula to blastocyst stage and is epigenetically maintained during subsequent mitotic cell divisions (Deng et al. 2014). Autosomal genes can also undergo random monoallelic expression, with initial descriptions of its occurrence in immune genes (Mostoslavsky et al. 2001) and the olfactory receptors (Chess et al. 1994), providing cellular diversity within these systems. Recent high-throughput allelic expression profiling of human clonal lymphoblastoid, fibroblast, and neural stem cells has shown that stochastic allelic expression is more prevalent than previously thought, affecting up to 10% of autosomal expressed transcripts across a diverse range of cell types (Gimelbrant et al. 2007; Chess 2012; Jeffries et al. 2012; Li et al. 2012; Zwemer et al. 2012). This process has also been observed in mouse, with a significant overlap in loci characterized by random monoallelic expression across species (Zwemer et al. 2012). The cellular diversity resulting from such stochastic monoallelic gene expression is likely to result in considerable clonal level phenotypic heterogeneity within tissues.

Although complex multiple-promoter regulated expression drives the stochastic process of allelic choice in a limited number of genes, for example the immune gene *Ly49* and the protocadherin clusters alpha and gamma (Ensminger and Chess 2004; Chess 2005), the mechanisms driving most cases of autosomal random monoallelic expression are not known. It has been shown that while biallelic genes are synchronously replicated in S-phase, random monoallelically expressed genes show asynchronous replication, even before the activation of transcription (Kitsberg et al. 1993; Simon et al. 1999). Epigenetic processes such as DNA methylation and histone modifications are also associated with this form of transcriptional control (Jeffries et al. 2012; Nag et al. 2013). While increased DNA methylation at the gene promoter is associated with monoallelic gene expression (Gendrel et al. 2014), DNA methylation on its own may not be sufficient for allelic expression control (Eckersley-Maslin et al. 2014; Gendrel et al. 2014).

Stochastic monoallelic loci are distributed across the genome and encompass most functional gene ontologies, although some studies indicate that they are enriched among cell surface molecules (Gimelbrant et al. 2007; Jeffries et al. 2012). Two recent studies examined the developmental patterns of random monoallelic expression using clonally expanded embryonic stem cells (ES) and neural progenitor cells from outcrossed mice (Eckersley-Maslin et al. 2014; Gendrel et al. 2014). Approximately 0.5% of transcribed genes in ES cells were characterized by random monoallelic expression, compared to 2%–3% of expressed genes in derived neural progenitor cells. There was little overlap in the specific monoallelic genes between ES cells and neural progenitor cells, although there was overlap between the neural progenitor cells from the two studies (Eckersley-Maslin et al. 2014; Gendrel et al. 2014; Reinius and Sandberg 2015). Allelic expression has also been examined in human fibroblasts reprogrammed into induced pluripotent stem cells (iPSCs) (Tanaka et al. 2015). A bias or allelic preference was observed in a subset of genes during the intermediate stage of reprogramming, with biallelic expression restored when iPSC reprogramming is complete.

In this study, we examined the effects of epigenetic reprogramming on allelic expression decisions within a defined clonal human neural progenitor cell line. We first quantified the extent of random monoallelic expression and related this to DNA methylation in fetal-derived clonal neural progenitor cells. Subsequently, we assessed allelic patterns of gene expression after epigenetic reprogramming into a pluripotent state (Takahashi and Yamanaka 2006). Finally, the resulting iPSCs were committed back into a neural lineage, and clonal neural stem cells subsequently profiled. We show, for the first time in human cells, the dynamic nature of allelic expression patterns across pluripotent and lineage committed states.

## RESULTS

### Epigenetic reprogramming to induced pluripotency resets random allelic expression imbalance

To investigate the effect of epigenetic reprogramming to induced pluripotency on stochastic allelic expression, we profiled allelic expression patterns in three conditionally immortalized clonal human neural stem cells (proliferative state: SPC01, SPC04, and SPC06; and differentiated state: SPC01D, SPC04D, and SPC06D) and reprogrammed iPSC clones derived from one of these (SPC01). Two iPSC clones were profiled (iPS1 and iPS2) at “early” (6–10 passages) and “late” (20–22 passages) passages (see Supplemental Fig. S1 for an overview of our experimental design). Approximately 7000 autosomal genes contained informative (heterozygous) transcribed SNPs (see Materials and Methods) allowing us to make quantitative allelic expression measurements. Based on previous allelic expression studies (Pastinen et al. 2004; Serre et al. 2008; Lee et al. 2009; Jeffries et al. 2012) and X-chromosome inactivation profiles (Supplemental Fig. S2), we categorized the expression of genes as biallelic (BA), allelically skewed, and monoallelic (MA) according to the degree of allelic bias detected. A summary of the allelic expression patterns for all clonal lines profiled in this study is shown in Table 1A together with the changes in allelic expression between different developmental states shown in Table 1B–D. More detailed alternate isoform level results for each developmental state are shown in Supplemental File S1. We also identified a number of genes characterized by MA expression in one clone but BA expression in other clones of the same developmental state. We describe these as random allelic expression imbalanced genes (the term random monoallelic expression would be assigned if both alleles were detected in additional clones examined). The frequency of these genes is shown in Table 1, and lists of MA genes in Supplemental File S2.

**TABLE 1.**
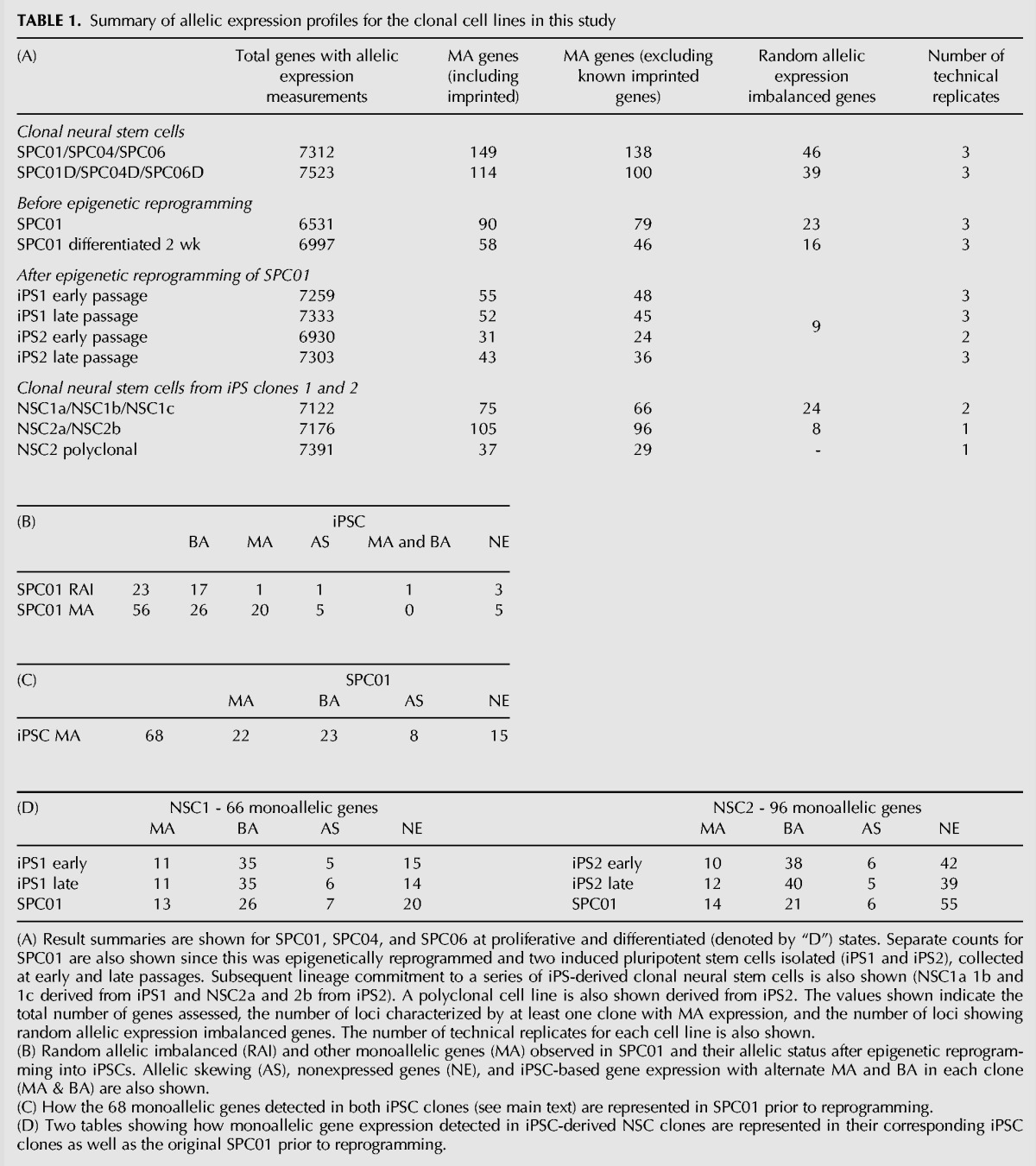
Summary of allelic expression profiles for the clonal cell lines in this study

**FIGURE 1.**
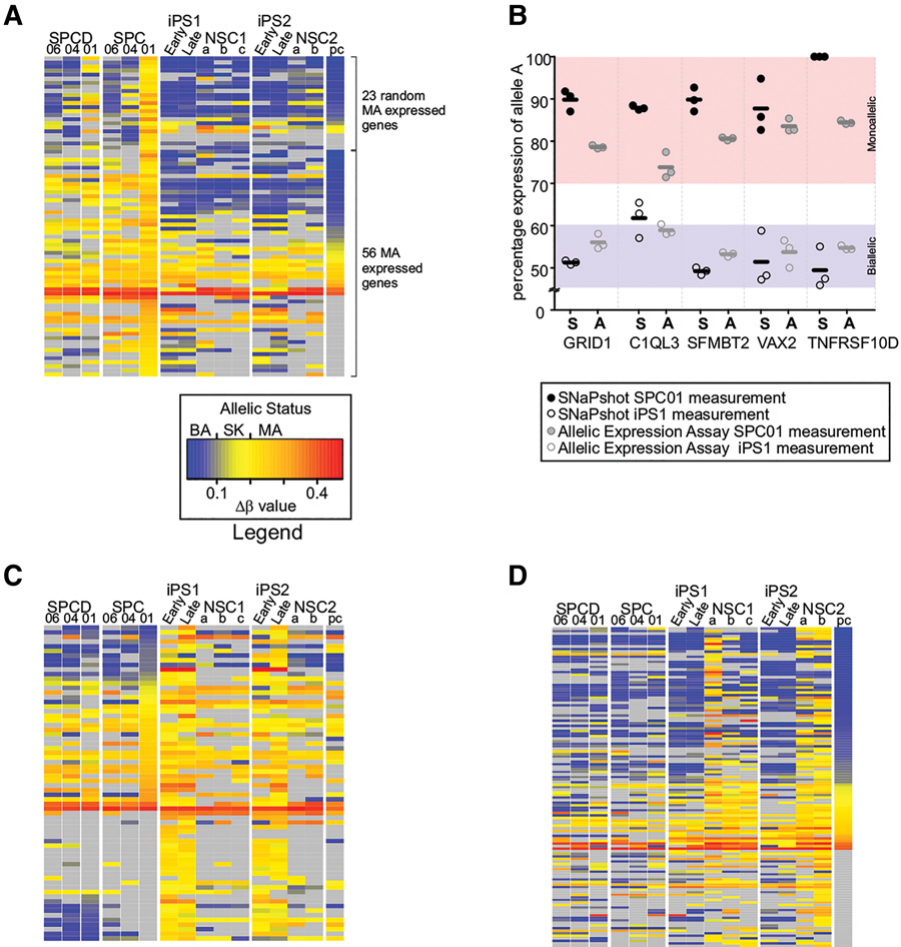
Changes in allelic expression are seen across different developmental states. (*A*) Allelic expression profiles for genes identified as monoallelically expressed in the SPC01 neural stem cell. Allelic expression is shown for SPC01, SPC04, and SPC06 at proliferative and differentiated states (denoted with “D”) as well as its resulting iPS clones at early and late passage (labeled “Early” and “Late”), together with iPS-derived clonal NSC's and a polyclonal cell isolate (labeled “pc”). Genes have been categorized into two groups—those positively identified as showing random allelic expression imbalance and those which only show MA expression in measured clones. The abbreviation SK indicates genes showing allelic skewing. (*B*) Validation of the resetting of monoallelic expression detected in SPC01 to a more biallelic form of allelic expression in iPSC clones. The percentage expression of the major expressed allele is shown in relation to the highlighted regions indicating monoallelic and biallelic expression. “S” denotes the SNaPshot primer extension assay results and “A” denotes the allelic expression results from the Illumina beadchip. (*C*) MA genes found in the epigenetically reprogrammed iPSCs. (*D*) MA genes identified in the iPSC-derived neural stem cells. See Supplemental Figure S3 for high-resolution heatmaps.

In the clonal neural stem cells, 138 nonimprinted autosomal genes (1.88% of total assessed genes) were characterized as demonstrating MA expression (i.e., allelic ratio >2.85-fold, see Materials and Methods), of which 46 (0.63% of total assessed genes) showed evidence of random allelic expression imbalance based on differing allelic expression profiles in sister clones. Our subsequent analyses focused primarily on the representative clone SPC01, which was characterized by 79 autosomal MA-expressed genes, not known to be genomically imprinted. By examination of the two sister clones for alternative allelic expression profiles, we conservatively estimate that 23 (29% of SPC01 expressed MA genes) show evidence of random allelic expression imbalance. After epigenetic reprogramming to induced pluripotency, 20 of these random allelic expression imbalanced genes were expressed at detectable levels in the resultant iPSC clones. Strikingly, 17 (85%) of these initial random allelic expression imbalanced loci showed clear BA expression after reprogramming (Table 1B; Fig. 1A). Allelic expression measurements were successfully validated by single nucleotide primer extension assays at all loci tested (Fig. 1B). While reprogramming involves a number of cell signaling pathways, the widespread epigenomic changes that occur during reprogramming (Koche et al. 2011; Polo et al. 2012) give support toward a role for epigenetic processes regulating random allelic expression imbalance.

An additional 56 MA gene loci detected in the SPC01 cell line could not be unequivocally identified as being characterized by random allelic expression imbalance because either the same allele was expressed in all three sister clones or expression was not reliably detected in all clones (see Fig. 1C). Examining their response to reprogramming revealed that 26 of the 56 genes (46%) reverted to BA expression in the iPSCs (Table 1B), while the remaining 30 transcripts were characterized by stable MA expression (20 genes/36%), allelic skewing (five genes/9%), or no detectable expression (five genes/9%) after epigenetic reprogramming. Therefore, the remodeling of epigenetic marks during reprogramming to a pluripotent developmental state appears to erase the MA expression status of most random allelic expression imbalanced genes and ∼50% of the additional 56 MA expressed genes that are likely to contain additional random allelic expression imbalanced genes. The genes that remain MA after reprogramming are likely to represent loci showing a resistance to epigenetic reprogramming (Chen et al. 2013a) or examples of genetically driven MA expression—i.e., *cis*-acting expression quantitative trait loci (eQTLs) (Ge et al. 2009). To investigate this further, we cross-referenced these genes to loci characterized by aberrant gene expression in iPS cells (Chin et al. 2009) and found no overlap. We also checked 841 genes mapping to known hotspots of aberrant epigenetic reprogramming defined by DNA methylation differences between iPSCs and embryonic stem cells (Lister et al. 2011). Five of these genes (*DPP6*, *FTCD*, *HEATR4*, *LRRC61*, and *MYH14*) were present in our list of 22 constant MA expressed genes. This represents about twice as many genes as would be expected by chance but does not represent a statistically significant enrichment (two-tailed Fisher's exact test *P*-value = 0.41). From this, we conclude that it is likely that the majority of MA expressing genes that fail to respond to the reprogramming actually represent examples of genetically driven MA expression.

We also examined an additional 23 random allelic expression imbalanced genes, characterized by BA expression in the SPC01 cell line but MA expression in either of the sister clonal lines (SPC04 or SPC06). Of these genes, 20 showed measurable expression in the iPS state, all with biallelic expression except *NLRP2*, which showed random allelic expression imbalance. After neuralization, the majority of the genes maintained BA expression apart from *NLRP2*, which showed MA expression, and *SYT3*, which was characterized by skewed allelic expression in two neural stem cell clones.

### Novel monoallelic gene expression in reprogrammed iPSCs

Allelic expression profiling of the two iPSC clones at early and late passage identified 68 genes (0.88% total genes assayed) characterized by MA expression in at least one iPSC clone (iPS1 early and late passage clones = 62 genes, iPS2 early and late passage clones = 39 genes, overlap between two IPSC clonal sets = 33 genes). Of these, 22 genes were characterized as being MA in the original SPC01 cell line before reprogramming, suggesting that their allelic expression status is resistant to epigenetic reprogramming or influenced by *cis*-acting genetic factors. The remaining 46 genes (0.59% of total genes assayed) potentially indicate de novo MA expression emerging at the pluripotent state since 50% of these (23 genes) were previously characterized by BA expression in SPC01 clones before reprogramming (in the proliferative or differentiated state), with the remaining genes showing clear allelic imbalance (eight genes/17.4%) or levels of expression below detection (15 genes/32.6%).

More detailed analysis of the individual clones and comparison of passage number highlighted nine genes (*CACNB2*, *DNAJA4*, *RBFOX1*, *LOC647946*, *MYH14*, *NLRP2*, *TCERG1L*, and *SYCP2L*) characterized by apparent random allelic expression imbalance as demonstrated by MA expression in one iPSC clone and BA expression in the other clone. One of these loci, *RBFOX1*, is an RNA-binding protein associated with autism (Sebat et al. 2007). This gene shows similar random allelic expression imbalance in a previous allelic expression study examining iPSCs and differentiated neurons (Lin et al. 2012). Furthermore, two genes, *ZNF528* and *PKIB*, while consistently characterized by BA expression in iPS1 and early passage iPS2 cells, converted to MA expression in late passage iPS2 clones. Interestingly, the MA expression of *PKIB* was maintained in the resulting iPSC-derived NSC2 (both clonal and polyclonal), whereas *ZNF528* reverted back to BA expression.

### Neuralization reestablishes random allelic expression imbalance

The majority (85%) of random allelic expression imbalanced genes identified in the original SPC01 neural stem cells reverted to BA expression after epigenetic reprogramming to induced pluripotency. To explore what happens when these iPSCs are recommitted to a more restricted neural progenitor cell lineage, we neuralized two iPSC clones using a dual SMAD inhibition method (see Materials and Methods) to produce rosette-like neural stem cells (Chambers et al. 2009). We subsequently sequenced cDNA for previously identified random allelic expression imbalanced genes, identifying BA expression signatures in the iPSC-derived neural stem cells (Supplemental Fig. S4) as would be expected since this population of neural cells is polyclonal, derived from multiple individual neuralized cells.

We subsequently isolated clonal cell lines by dilution plating of this polyclonal population, and cultured the surviving colonies to a sufficient density for RNA isolation and profiling. Five neural stem cell clones were isolated in total (three from one iPS1 clone [termed NSC1] and two from a second iPS2 clone [termed NSC2]) in addition to the polyclonal population sample. Approximately 7100 genes were informative for allelic expression profiling, of which 66 nonimprinted autosomal genes (0.93% of total assayed genes) were identified as being characterized by MA expression in at least one of the NSC1 clones. Ninety-six MA-expressed genes (1.34% of total assayed genes) were identified in the NSC2 clone set, although the lack of a technical replicate in this latter set may be one reason for the higher call rate. Examining the overlap between clone sets, 37 (58.7%) of 63 genes expressed in both clonal sets showed evidence of MA expression in both NSC1 and NSC2 clones. Many of these NSC1 and NSC2 genes represent novel MA loci and were not characterized by MA expression in the original SPC01 line. The limited gene ontology analyses we could undertake given the small number of input loci provided evidence for an overrepresentation of proteins integral to the plasma membrane (GO:0005887, *P* = 0.04, 1.97-fold enrichment), reflecting the patterns seen in the original clonal neural stem cell before reprogramming in addition to neural stem cells from other brain regions (Jeffries et al. 2012) and clonal lymphoblastoid cells (Gimelbrant et al. 2007).

Using the clonal results, we estimated the frequency of random allelic expression imbalance identifying alternate allelic expression patterns across clones. Individually, 24 random allelic expression imbalanced genes were identified in the NSC1 clones (36.4% of total MA genes identified within the three clones) and eight in NSC2 (8.3% of total MA genes from two clones). Combining NSC1 and NSC2 to give five clones showed 7334 total genes expressed, with 126 genes (1.71%) showing at least one clone with MA expression and 48 of these (0.65% of total expressed genes) displaying random allelic expression imbalance. An alternate approach to estimate the frequency of random allelic expression imbalance is to examine a polyclonal population in combination with clonal cells. Since random allelic expression imbalance choice appears to be a stochastic process, measurements in polyclonal populations are expected to show approximately equal quantities of both alleles, i.e., apparent BA expression. We found that 42 of the 48 (88%) random allelic expression imbalanced genes identified in the five clones of NSC1 and NSC2 were characterized by a BA signal when examined in polyclonal neural stem cells. The polyclonal neural stem cell showed measurable expression for 88 of the 126 MA expressed genes identified in the NSC1 and NSC2 clones. From these, 62 genes (70.5%) were characterized by BA expression in the polyclonal cell line, indicating a higher frequency of 0.84% of total expressed genes showing random allelic expression imbalance frequency compared to our estimate of 0.65% using clonal lines alone. Seventeen genes were also identified as being MA expressed (19.3%) in the polyclonal line, with nine genes with allelic skewing (10.2%), presumably influenced by *cis*-acting genetic effects. Based on our data, we conclude that lineage commitment commits approximately 1.7% of genes to MA expression, of which between 0.65% and 0.84% of expressed genes are characterized by random allelic expression imbalance.

### DNA methylation correlates with allelic status

**FIGURE 2.**
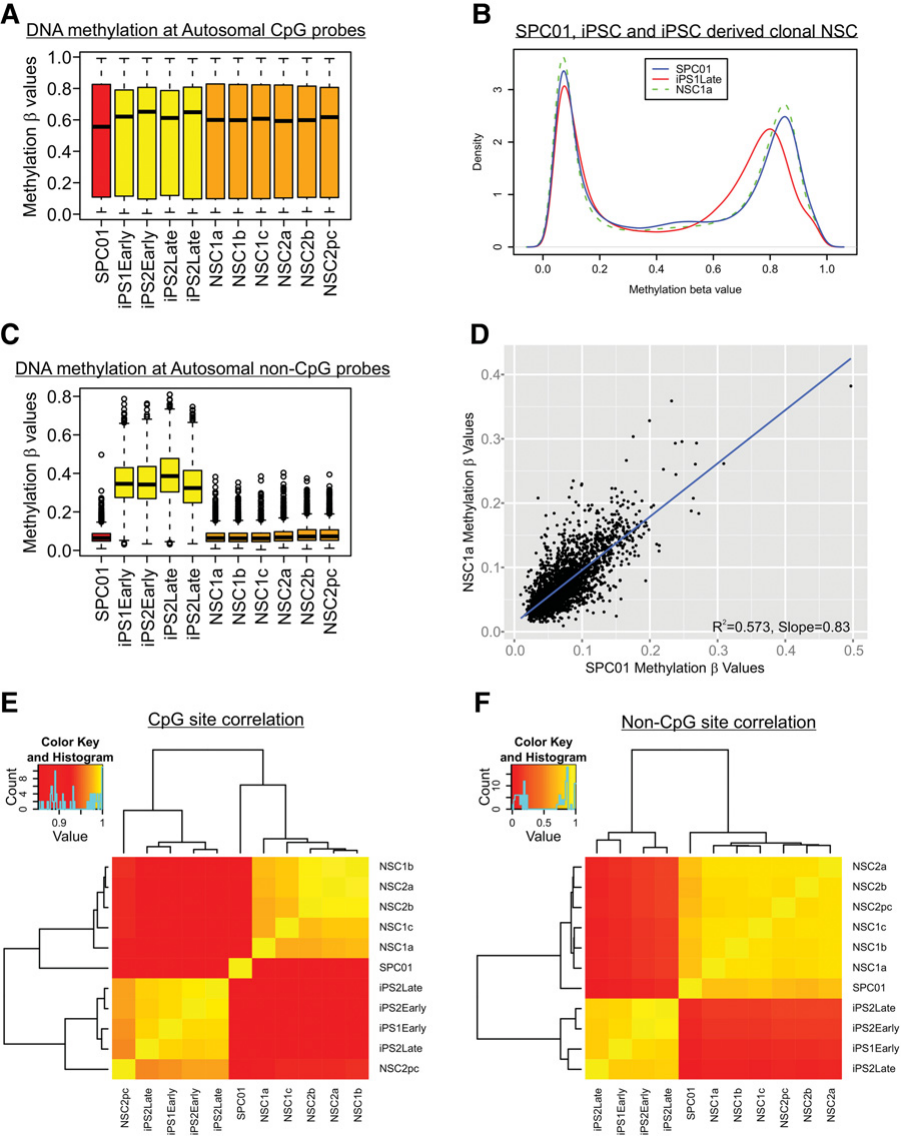
Monoallelic gene expression is associated with elevated levels of DNA methylation. Autosomal DNA methylation profiles are shown for SPC01 neural stem cell, induced pluripotent stem cells (iPSs), and iPSC-derived neural stem cells (NSCs). (*A*) DNA methylation levels at autosomal CpG sites. (*B*) The methylation β-value distribution changes for autosomal CpG sites in the different developmental cell types studied. (*C*) Levels of DNA methylation at 2902 autosomal non-CpG sites. Significant increases in DNA methylation can be seen in iPS cells when compared to the original donor SPC01 neural stem cell and also resulting iPS-derived NSCs. (*D*) Scatterplot illustrating similar status of non-CpG DNA methylation of iPS-derived NSCs versus the original SPC01 NSC. (*E*) Heatmap of DNA methylation values for autosomal CpG sites together with hierarchical clustering of each sample. (*F*) Heatmap of DNA methylation values for non-CpG sites. Additional DNA methylation data are shown in Supplemental Figure S5.

Global patterns of DNA methylation (Fig. 2A,B) show only modest changes associated with developmental state, although we observe large changes in DNA methylation at specific CpG sites; 40,425 CpGs of 415,863 were characterized by >30% (Δβ > 0.3) differences in DNA methylation between SPC01 and iPSCs before and after reprogramming. Increased levels of non-CpG site DNA methylation were also found during the pluripotent iPSC stage (Fig. 2C,D) compared to neural stem cells before and after reprogramming. Hierarchical clustering based on CpG and non-CpG sites distinguished the different clonal developmental states used in this study (Fig. 2E,F). Overall, the DNA methylation changes described are in agreement with expected changes reported from a previous study examining multiple tissue types and their reprogrammed pluripotent counterparts (Nishino et al. 2011).

Building on our previous evidence for increased levels of DNA methylation associated with random MA expressed genes (Jeffries et al. 2012), we compared DNA methylation in iPSC-derived NSC clones showing MA expression to clones showing BA expression, identifying dramatic differences at numerous loci. For example, *TNFRSF10D*, which we previously reported as showing random allelic expression imbalance (Jeffries et al. 2012), is significantly hypomethylated in reprogrammed iPSC compared to the MA expressing SPC01 cell line across all probes associated with an exon spanning CpG island (average DNA methylation difference = 19.9%, paired *t*-test *P*-value = 0.015) (Supplemental Fig. S6). Lineage commitment associated with skewed allelic expression is characterized by increased DNA methylation at this locus, whereas a clone with no detectable expression is characterized by even higher DNA methylation levels.

**FIGURE 3.**
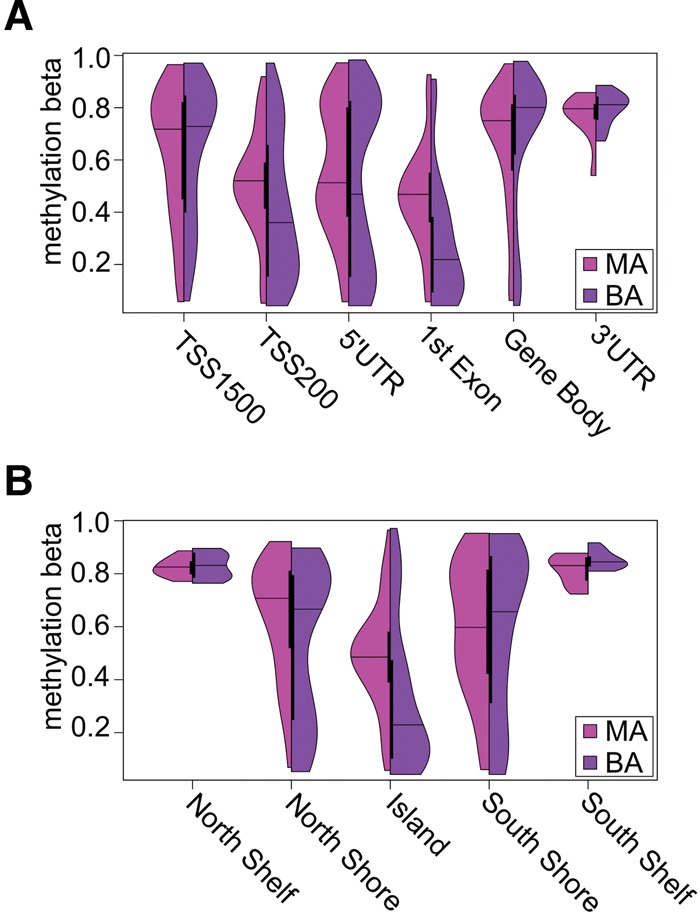
Monoallelic gene expressions are associated with increased DNA methylation at the transcriptional start site and CpG island sequences. Violin plots illustrate the distribution of DNA methylation levels at autosomal CpG sites for random allelic expression imbalanced genes in iPS-derived neural stem cell NSC1 clones expressed as either MA (purple) or BA (cyan). (*A*) DNA methylation levels at annotated regions 1500 and 200 bp from the transcriptional start site (TSS1500 and TSS200), 5′ and 3′ untranslated regions (5′UTR and 3′UTR), regions associated with the first exon (1st Exon) and those within the gene body (Body). Significantly increased DNA methylation was found in MA expressing clones at the TSS200 region (Wilcoxon rank sum *P*-value = 0.00601) and 1st Exon (*P* = 3.726 × 10^−7^). The gene body also showed increased DNA methylation in BA clones (*P* = 0.003458) (*B*) CpG Island features show significantly increased DNA methylation was associated with MA expressed genes within CpG Islands (*P* = 4.799 × 10^−10^) rather than the shores and shelves.

Overall, three broad classes of genic region were characterized by differences in DNA methylation within MA expressing clones compared to BA clones (Fig. 3; Supplemental Table S1). Increased DNA methylation was found in MA expression clones at regions ∼200 bp upstream of the transcriptional start site (paired Wilcoxon rank sum test *P*-value = 0.006), indicative of differential methylation at the gene promoter, and similar increased expression at the first exon (paired Wilcoxon rank sum test *P*-value = 3.726 × 10^−7^). The gene body, in contrast, showed decreased DNA methylation in MA expressing clones (paired Wilcoxon rank sum test *P*-value = 0.003458). These observations of increased DNA methylation at the promoter and decreased levels within the gene body are interesting given the repressed transcriptional activity of MA expressed genes (Jones 2012). We also examined DNA methylation across annotated CpG island features. CpG island shelves and shores showed no statistically significant difference. However, more dramatic differences in DNA methylation were observed within actual CpG islands, with significantly increased DNA methylation associated with MA expression (paired Wilcoxon rank sum test *P*-value = 4.799 × 10^−10^).

Previous studies have also shown an association with other epigenetic signatures (Jeffries et al. 2012; Nag et al. 2013; Eckersley-Maslin et al. 2014; Gendrel et al. 2014). To investigate this further, we utilized reference human epigenomes (Roadmap Epigenomics Consortium et al. 2015) for human ES cells (H1 and H9) as well as ES-derived neural progenitor cells. Gene loci showing monoallelic expression or random allelic imbalance in our iPS-derived NSCs from this study showed significantly decreased levels of the active chromatin marks H3K4me3 and DNase I hypersensitivity sites (Supplemental Fig. S7) in both pluripotent ES cell and neural progenitor cell reference epigenomes. Conversely, significantly increased levels of the repressive H3K27me3 were observed for these genes when compared to biallelic gene loci. To investigate whether developmental-specific differences were present, we selected gene loci showing biallelic expression in iPS1 and concomitant monoallelic expression in at least one of the iPS1-derived NSC1 clones (Supplemental Fig. S8). Increase levels of H3K4me3 were found in both the H1 and H9 ES cell lines when compared to the neural progenitor derivatives (Wilcoxon rank sum *P*-value <2.2 × 10^−16^). However, conflicting results were found for the repressive H3K27me3 modification, with H1 ES cells showing higher levels than their neural progenitor counterparts compared to H9 neural progenitors showing higher levels than their original ES cell status (Wilcoxon rank sum *P*-value <2.2 × 10^−16^). No differences were observed at DNase I hypersensitivity sites (Wilcoxon rank sum *P*-value = 0.78).

## DISCUSSION

The aim of this study was to assess the effects of induced pluripotency on genes characterized by random allelic expression imbalance. We show that the majority of random allelic expression imbalanced genes with initial MA expression were reset to a BA status when reprogrammed. After lineage commitment back into a neural stem cell state, MA expression was reestablished in some of the original MA genes, in addition to many new genes that had been BA-expressed prior to reprogramming. We also observed some de novo MA expression arising in the iPSC clones, perhaps representing partial commitment to random allelic expression imbalance or simply reflecting a dynamic allele-specific transcriptome. Similar de novo allelic expression bias was also noted in another study examining human iPSC reprogramming stages (Tanaka et al. 2015) and murine ES cells (Eckersley-Maslin et al. 2014; Gendrel et al. 2014).

The primary cause of monoallelic expression was originally thought to be through genomic rearrangements based on early cloning experiments of mature lymphocytes which resulted in monoclonal mice containing the same rearranged t-cell receptor gene in all tissues (Hochedlinger and Jaenisch 2002). However, nuclear transfer experiments of post-mitotic olfactory neurons proved otherwise, showing allelic expression status was reset upon nuclear transfer (Eggan et al. 2004; Li et al. 2004) resulting in cloned mice with a full repertoire of olfactory receptors. Our experiment builds on this and shows widespread resetting of monoallelic expression in genes undergoing random allelic expression imbalance/random monoallelic expression control after reprogramming to pluripotency. Previous to this work, two papers examining murine ES cells and derived neural progenitor cells detected increased levels of random monoallelic/imbalanced expression upon differentiation (0.5% genes in ES cells versus 3% in neural progenitor cells). Our study shows a lower estimate of random allelic imbalanced expression in analogous iPSC cells and human neural progenitor cells, which may reflect the lower number of clones examined.

If the pluripotency and lineage committed results correctly extrapolate in vivo, it suggests allelic expression assignment occurs during either gastrulation or neurulation. An overrepresentation of genes encoding cell surface/cell membrane proteins was found among the MA-expressing loci, agreeing with previous studies (Gimelbrant et al. 2007; Jeffries et al. 2012). Cell surface diversity may therefore be a factor driven by random allelic expression imbalance, allowing alternate isoforms that may encode functional differences to be expressed in a subset of cells. This process would also provide a mechanism to support dosage variation, a phenomenon previously associated with monoallelic expression (Gimelbrant et al. 2007; Jeffries et al. 2012; Li et al. 2012). This may have profound implications for tissues such as the brain where cell identity and cell migration are highly dependent on receptor–ligand or receptor–morphogen interactions. As previously hypothesized, it may also contribute to disease discordance observed in monozygotic twin-pairs (Jeffries et al. 2013; Oey et al. 2015).

Recent single cell gene expression analyses have shown evidence for widespread monoallelic expression, although this is often dynamic in nature, presumably from the stochastic burst-like nature of transcription (Reinius and Sandberg 2015). However, mitotically stable random monoallelic expression (and allelic expression imbalance), as detected in clonal cell line studies such as in this study, is more likely mediated by epigenomic modifications. We and others find differential promoter level DNA methylation, a reduction of active chromatin signatures and increased repressive histone modifications associated with many random monoallelic expressed loci (Jeffries et al. 2012; Eckersley-Maslin et al. 2014; Gendrel et al. 2014). However, use of 5-azacytidine to deplete cells of DNA methylation marks failed to alter MA expression in examined candidate genes (Eckersley-Maslin et al. 2014; Gendrel et al. 2014). This observation has some similarities to a study in mouse embryonic fibroblasts which examined imprinted genes after 5-azacytidine exposure (El Kharroubi et al. 2001). Exposure altered allelic expression in some imprinted genes but failed to have an impact on other imprinted gene loci (El Kharroubi et al. 2001), indicating differences in allelic regulation mechanisms between some of the imprinted MA expressed genes. It is uncertain whether random monoallelic expressed genes throughout the genome are regulated in the same way or have differences in control mechanisms. Regardless, these data indicate that an additional layer of epigenetic control, such as histone tail modifications or antisense RNA, may play a role in regulating monoallelic expression.

In conclusion, we provide evidence to support the notion that random or stochastic MA expression is a form of transcriptional control, which is set early in development during lineage commitment. The reprogramming of cells to an embryonic stem-cell-like state erases this form of transcriptional control but directed commitment to a neural stem cell lineage promotes its reassignment at a number of genes. Specific loci may show an inherent susceptibility to random allelic expression imbalance, with genes encoding cell surface molecules being particularly affected by this form of transcriptional control. Increased DNA methylation at the gene promoter, particularly across CpG Islands, is associated with monoallelic expressing clones and may play a key role in maintaining the repression of the inactive allele.

## MATERIALS AND METHODS

### Cell culture and derivation of induced pluripotent stem cells and neural stem cells

Conditionally immortalized human neural stem cell lines were derived from 10-wk-old fetal cervical spinal cord as described in Cocks et al. (2013). Three clones were used in this study (SPC01, SPC04, and SPC06) in their proliferative state, or following 2 wk of neural differentiation in culture (SPC01D, SPC04D, and SPC06D), which gives rise to a population of neurons and glia. The SPC01 cell line was epigenetically reprogrammed using a lentiviral vector to express *OCT4*, *SOX2*, *KLF4*, and *c-MYC*. Clones were isolated and pluripotency checked through the gene expression-based PluriTest (Müller et al. 2011) and immunocytochemistry for the pluripotency markers (Supplemental Fig. S9). Two iPS clones were carried forward in this study (iPS1 and iPS2), with early (6–10) and late (20–22) passage clones collected for analysis. Neural stem cells were also derived from the two iPSC clones using a dual SMAD inhibition procedure as described by Chambers et al. (2009). Clones were isolated through serial dilution into 96-well plates of single cells followed by expansion up to six-well plates for RNA and DNA harvesting. Expansion and subsequent profiling of two replicate wells was possible with NSC1 clones, although only one well was available for the NSC2 clones. This may result in higher incidence of false positives in the NSC2 clones.

### Nucleic acid isolation

RNA was collected and extracted using TRIzol (Life Technologies) followed by DNase treatment (DNA Turbo free, Life Technologies). DNase-treated RNA quality was assessed with the Agilent Bioanalyser (RIN > 9) and quantitative PCR on 200 ng of RNA to ensure no remaining genomic DNA. One microgram of RNA was converted to cDNA using random hexamers and Superscript III reverse transcriptase (Life Technologies) at 42°C for 2 h. Genomic DNA was extracted from cell pellets by incubation in sodium chloride-Tris-EDTA buffer containing 0.5% SDS with RNaseA (10 µg/mL) for 45 min at 37°C followed by Proteinase K (0.2 mg/mL) addition and further incubation at 50°C for 120 min. Phenol/chloroform extraction was then performed.

### Allelic expression analysis

We carried out a genome-wide allelic expression analysis using methods previously described in Jeffries et al. (2012). Briefly, the Illumina HumanOmniExpress-12 SNP beadchip was used to assess allelic expression using 250 ng of genomic DNA and cDNA equivalent of 200 ng of RNA. The resulting cDNA and gDNA results were separately quantile normalized across both channels before analysis. Any probes where the sum of X + Y (representing the scanned intensity for each allele) exceeded 750 were included in the analysis. Those below 750 were excluded as background signal noise. A β value of X/(X + Y) was then calculated to provide a quantitative scale of allelic expression rather than a binary assignment from standard genotyping software. The difference or Δβ value between cDNA measurements versus genomic DNA heterozygous SNPs was then used to calculate allelic expression at SNP probes, and the mean absolute Δβ value of all informative SNPs for RefSeq gene results. Intronic SNPs with sufficient intensity above background signal were included in the analysis since they also provide accurate allelic expression measurements (Pastinen et al. 2004; Gimelbrant et al. 2007; Serre et al. 2008). Allelic expression assignments were made according to the degree of allelic imbalance detected. MA expression is indicated by a 2.85 or greater difference in allele expression ratios (Δβ > 0.2), allelic expression skewing as a 1.5–2.85 (Δβ > 0.1 and Δβ < 0.2), and biallelic expression (BA) for those genes with a 1.0–1.5 ratio between alleles (Δβ < 0.1). The Δβ SNP probe distribution for autosomes compared to the X chromosome (containing a high number of inactivated/monoallelic expressed genes) is shown in Supplemental Figure S2 and highlights the use of Δβ > 0.2 being an appropriate threshold for MA expression. A penalty weighting score was also applied to minimize bias of large transcripts, described in more detail in Jeffries et al. (2012). Transcript-based analysis was performed in an isoform-specific manner. Known imprinted genes, as identified through geneimprint (http://geneimprint.com), catalog of imprinted genes (http://gc.otago.ac.nz) and a study by Morcos et al. (2011), were excluded from the primary analysis although are described in Supplemental Figure S10. Similarly, chromosome X status is described in Supplemental Figure S11. The α and γ protocadherins were detected as showing random monoallelic expression in this study but were removed from analysis to avoid bias based on their overlapping genome annotations. The lack of a technical cell culture well replicate in the NSC2 set is likely to account for the increased number of genes detected in this sample set (see Materials and Methods). All statistics were performed in R statistical environment 3.0.2 (R Development Core Team 2005) and gene ontology enrichment analysis was performed using DAVID (http://david.abcc.ncifcrf.gov/). Analyses looking for the presence of constitutive MA gene expression within possible regions of abberant reprogramming were performed using defined gene lists from Chin et al. (2009) and genomic regions from Lister et al. (2011), the latter of which were mapped to genes using GALAXY (https://usegalaxy.org). DNA sequence analyses were carried out using EpiGraph (http://epigraph.mpi-inf.mpg.de).

### Validation of allelic expression biases

Single-nucleotide primer extension analysis (SNaPshot, Applied Biosystems) was used to validate allelic expression biases identified using the SNP array. PCR amplification was performed on three genomic DNA samples and three independent reverse transcribed cDNA samples. Four microliters of each PCR reaction were electrophoresced and visualized on agarose gels to confirm specific amplification. Five microliters of PCR reactions were then treated with 1 U shrimp alkaline phosphatase (GE Healthcare) and 2 U Exonuclease I (New England Biolabs) at 37°C for 45 min and then at 85°C for 15 min prior to primer extension reactions. Primer extension reactions were then carried out on the treated PCR products in a total volume of 10 µL, containing 2 µL treated PCR product, 1.25 µL SNaPshot reagent (Applied Biosystems), 5.75 µL H_2_O and 1 pM extension primer. Primer extension thermocycling conditions consisted of an initial step of 95°C for 2 min, followed by 30 cycles of 95°C for 5 sec, 43°C for 5 sec and 60°C for 5 sec. Aliquots of 2 µL SNaPshot reaction product were combined with 8 µL Hi-Di formamide (Applied Biosystems) and electrophoresced on a 3130 Genetic Analyzer (Applied Biosystems). Peak heights of allele-specific extended primers were determined using GeneMarker software (SoftGenetics) and the ratio of the two peak heights calculated for each reaction to determine a β-value similar to the allelic expression assessments from the genome-wide allelic expression profiling.

### Epigenetic analysis

For DNA methylation analyses, genomic DNA (500 ng) from each sample was treated in duplicate with sodium bisulfite using the Zymo EZ DNA Methylation-Lightning Kit (Zymo Research). Genome-wide DNA methylation was quantified using the Illumina Infinium HumanMethylation450 BeadChip (Illumina) and scanned on the HiScan System (Illumina). Illumina GenomeStudio software (Illumina) was used to extract signal intensities for each probe, generating a final report that was imported into the R statistical environment 3.0.2 (R Development Core Team 2005) using the *methylumi* and *minfi* packages (http://bioconductor.org/packages). Data quality control and preprocessing were performed using the *wateRmelon* package as described previously (Pidsley et al. 2013). Cross hybridizing probes (Blair and Price 2012; Chen et al. 2013b) were removed from the analysis.

For our analysis of histone modifications and DNase I hypersensitivity sites, data generated from H1 and H9 ES cells as well as H1- and H9-derived neural progenitor cells using ChIP-seq and DNase I hypersentivity site mapping experiments were derived from release 9 of the preprocessed reference human epigenome database (http://egg2.wustl.edu/roadmap/web_portal/) generated by the NIH Roadmap Epigenomics Mapping Consortium (Roadmap Epigenomics Consortium et al. 2015). The corresponding −log_10_ Poisson *P*-values representing gene promoter regions (1000 bp upstream and 500 bp downstream of the transcriptional start site) were obtained from this resource and compared within R using boxplots and Wilcoxon rank sum tests.

## SUPPLEMENTAL MATERIAL

Supplemental material is available for this article.

## Supplementary Material

Supplemental Material
