# Supplementary material for: Erasure and reestablishment of random allelic expression imbalance after epigenetic reprogramming
Source: RNA. 2016 Oct;22(10):1620–30. doi: 10.1261/rna.058347.116 (PMC5029458; doi:10.1261/rna.058347.116)
Supplement: Supplemental Material [file supp_22_10_1620__index.html]

Erasure and reestablishment of random allelic expression imbalance after epigenetic reprogramming — Erasure and reestablishment of random allelic expression imbalance after epigenetic reprogramming — Supplemental Material 

# Erasure and reestablishment of random allelic expression imbalance after epigenetic reprogramming

## Supplemental Material

- Supplemental\_File1.xlsx
- Supplemental\_Figures\_and\_Tables.pdf
- Supplemental\_File2.xlsx
