# Supplementary material for: Erasure and reestablishment of random allelic expression imbalance after epigenetic reprogramming
Source: RNA. 2016 Oct;22(10):1620–30. doi: 10.1261/rna.058347.116 (PMC5029458; doi:10.1261/rna.058347.116)
Supplement: Supplemental Material [file supp_058347.116_Supplemental_Figures_and_Tables.pdf]

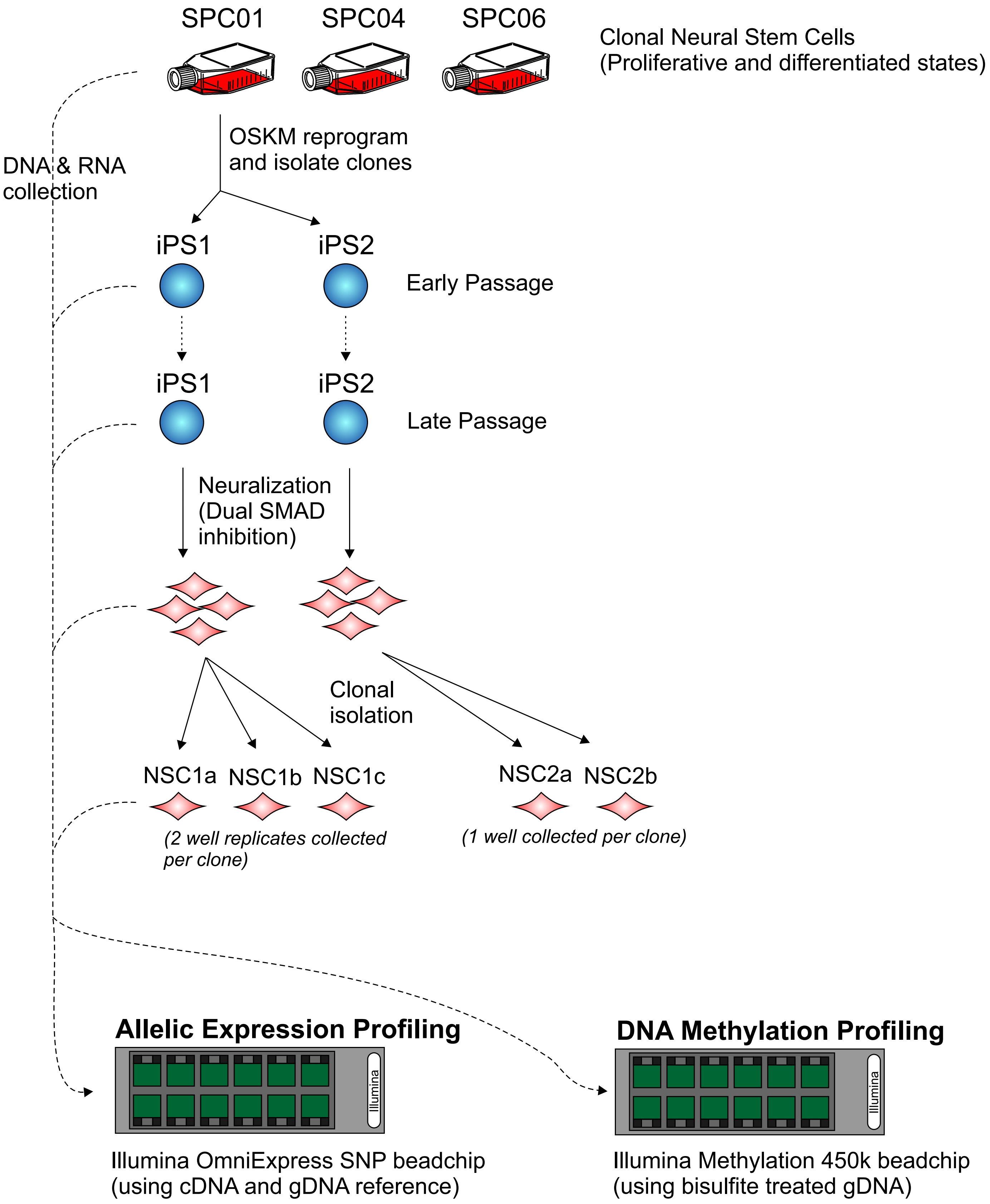

Figure S1. Illustration of the experimental design used in this study.

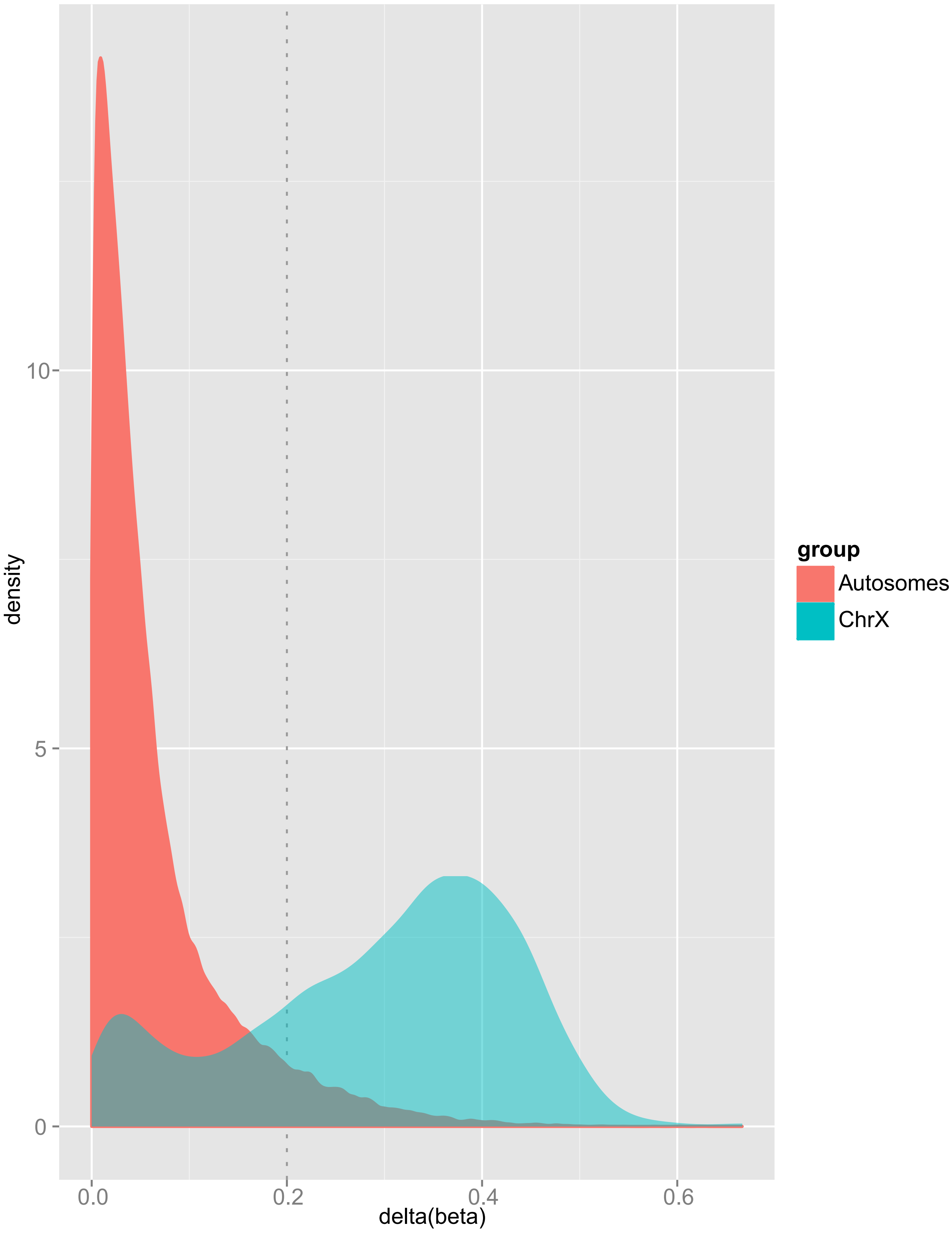

Figure S2. Probe level delta(beta) distribution for autosomes vs chromosome X. A small peak close to 0 on the chromosome X indicates genes escaping X inactivation. X inactivated genes are represented by a larger peak further along the delta(beta) scale. The dotted line signifies a delta(beta) of 0.2,which is within the secondary peak representing X-inactivated genes and is our chosen threshold for assigning monoallelic expression status to measured genes.

Monoallelic genes detected in SPC01

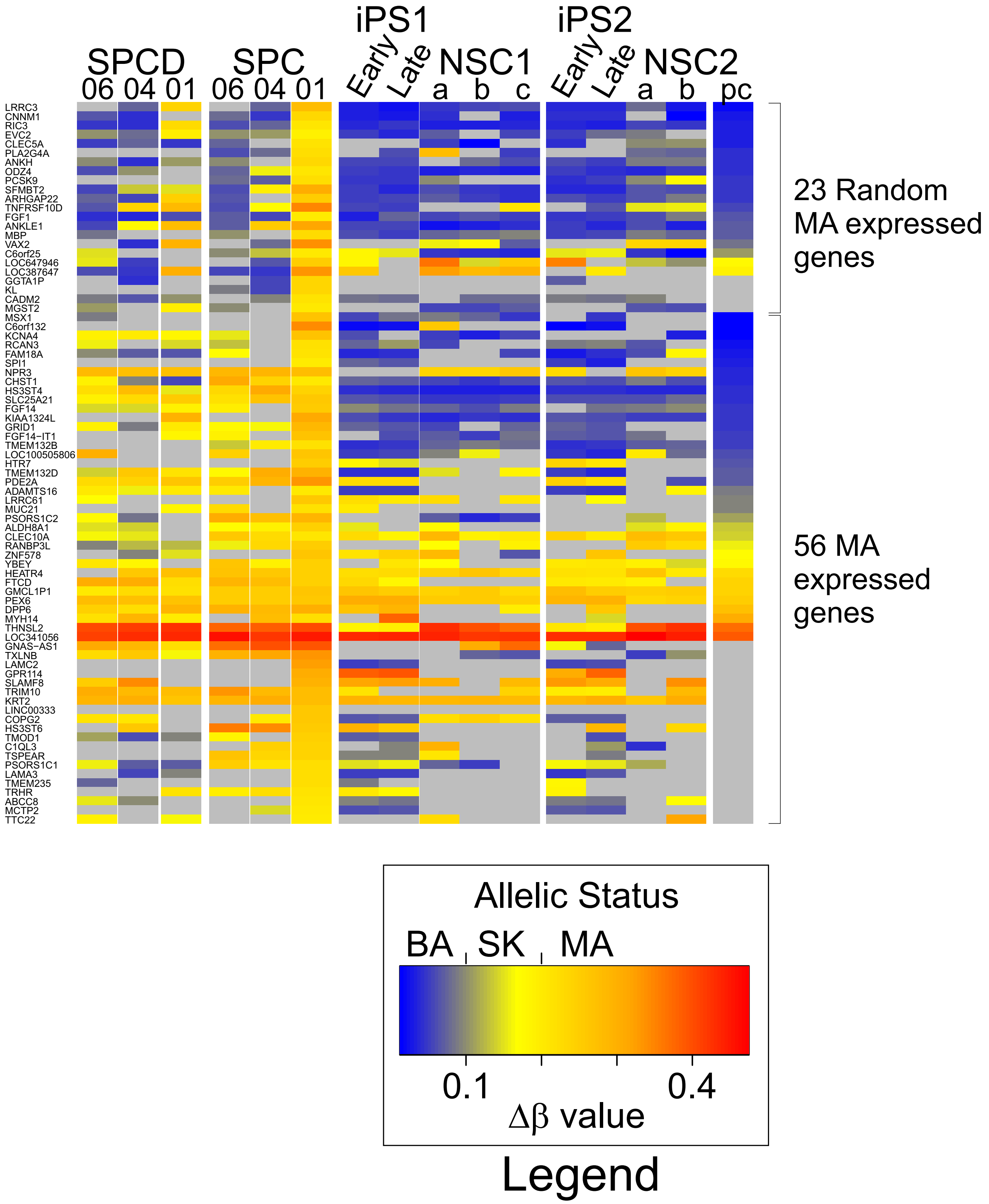

Figure S3. (a) Heatmap showing allelic expression measurements for genes originally detected as monoallelically expressed in SPC01. MA denotes monoallelic expression, SK denoted skewed allelic expression, BA denotes biallelic expression.

Monoallelic genes detected in iPSCs

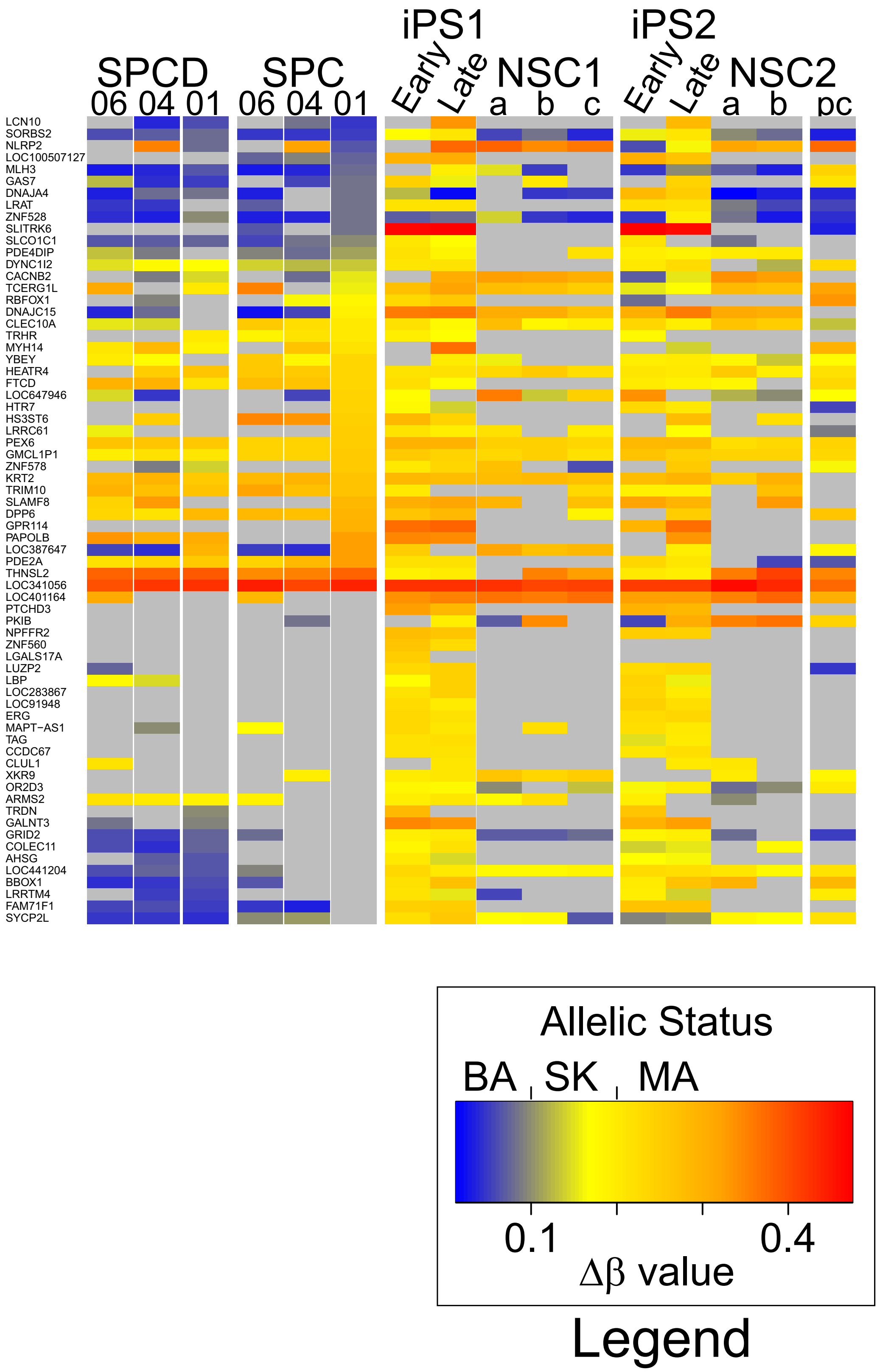

Figure S3. (b) Heatmap showing allelic expression measurements for genes originally detected as monoallelically expressed in iPSC clones

Monoallelic genes detected in NSCs

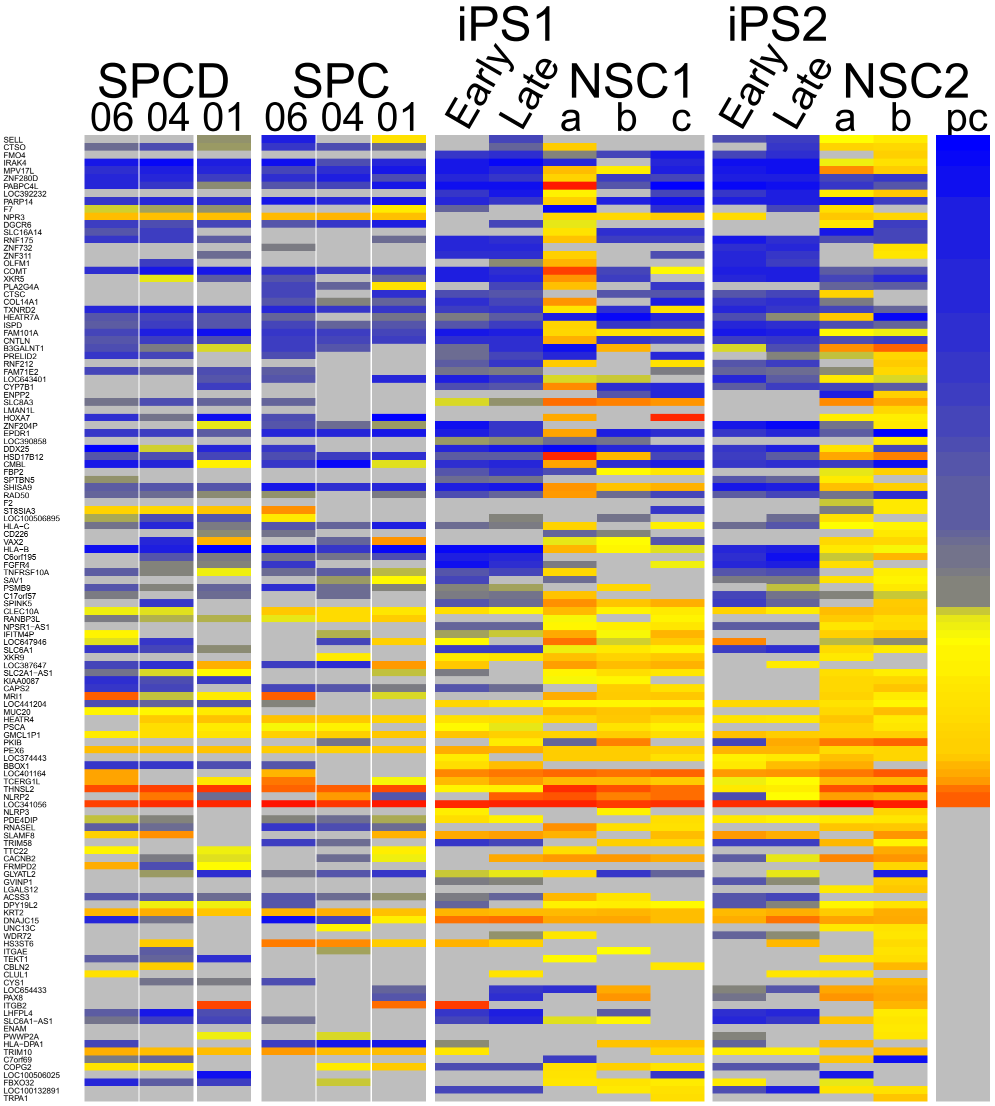

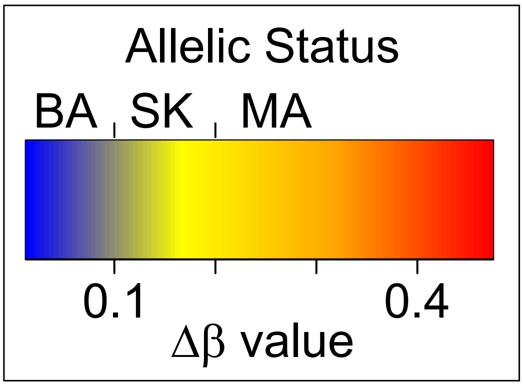

Legend

Figure S3. (c) Heatmap showing allelic expression measurements for genes originally detected as monoallelically expressed in iPSC derived neural stem cells (NSC1 and NSC2 clones)

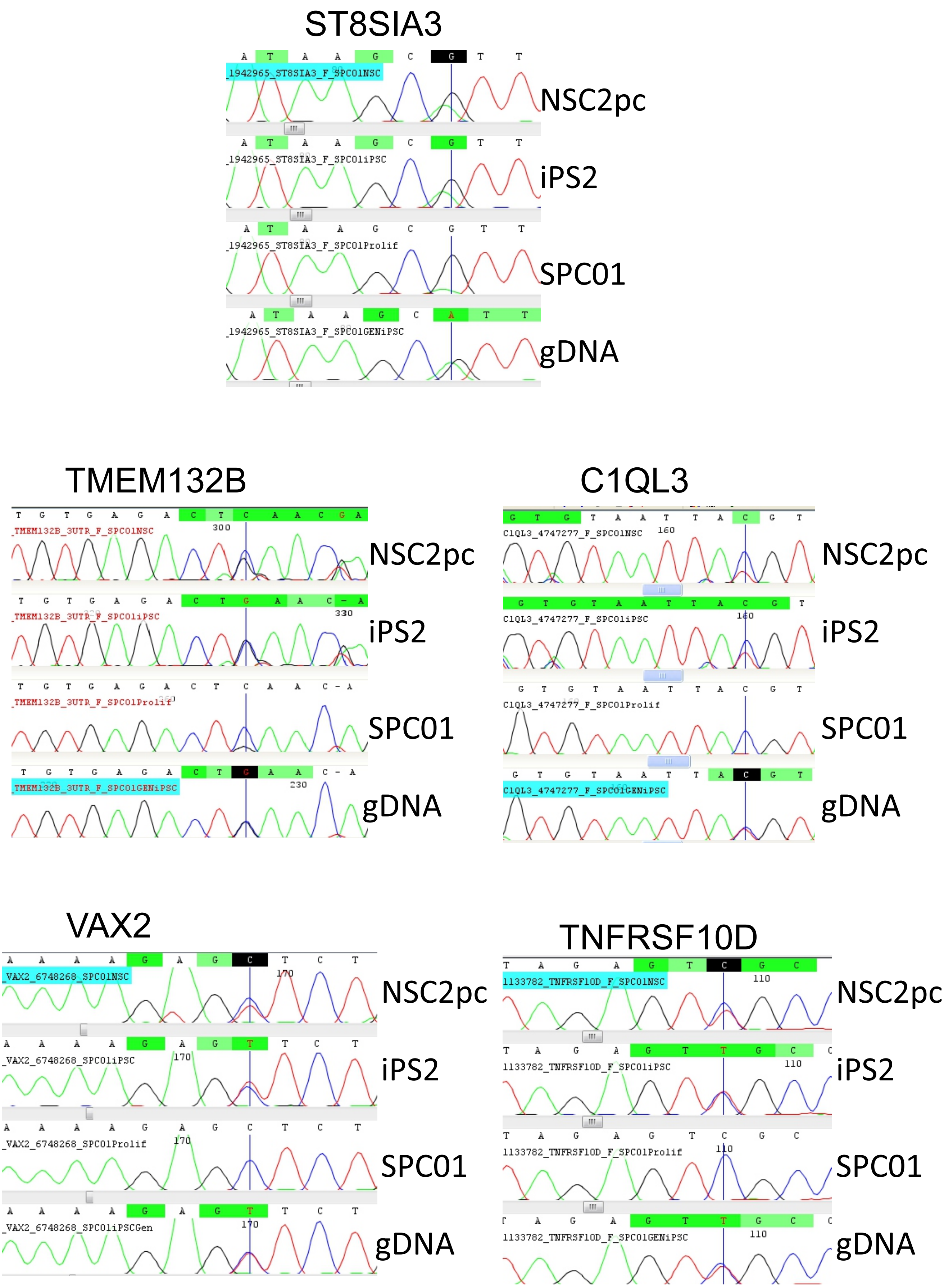

Figure S4. Sanger sequencing of gDNA to identify an informative heterozygous SNP and its expressed representation in SPC01, an iPS clone (reprogrammed SPC01), and a neural stem cell (NSC2pc) derived from the iPS2 cell. Each tested gene shows monoallelic expression in the SPC01 followed by reversion to biallelic expression in the iPS state. Biallelic expression is also shown in the NSCs and indicates a polyclonal population.

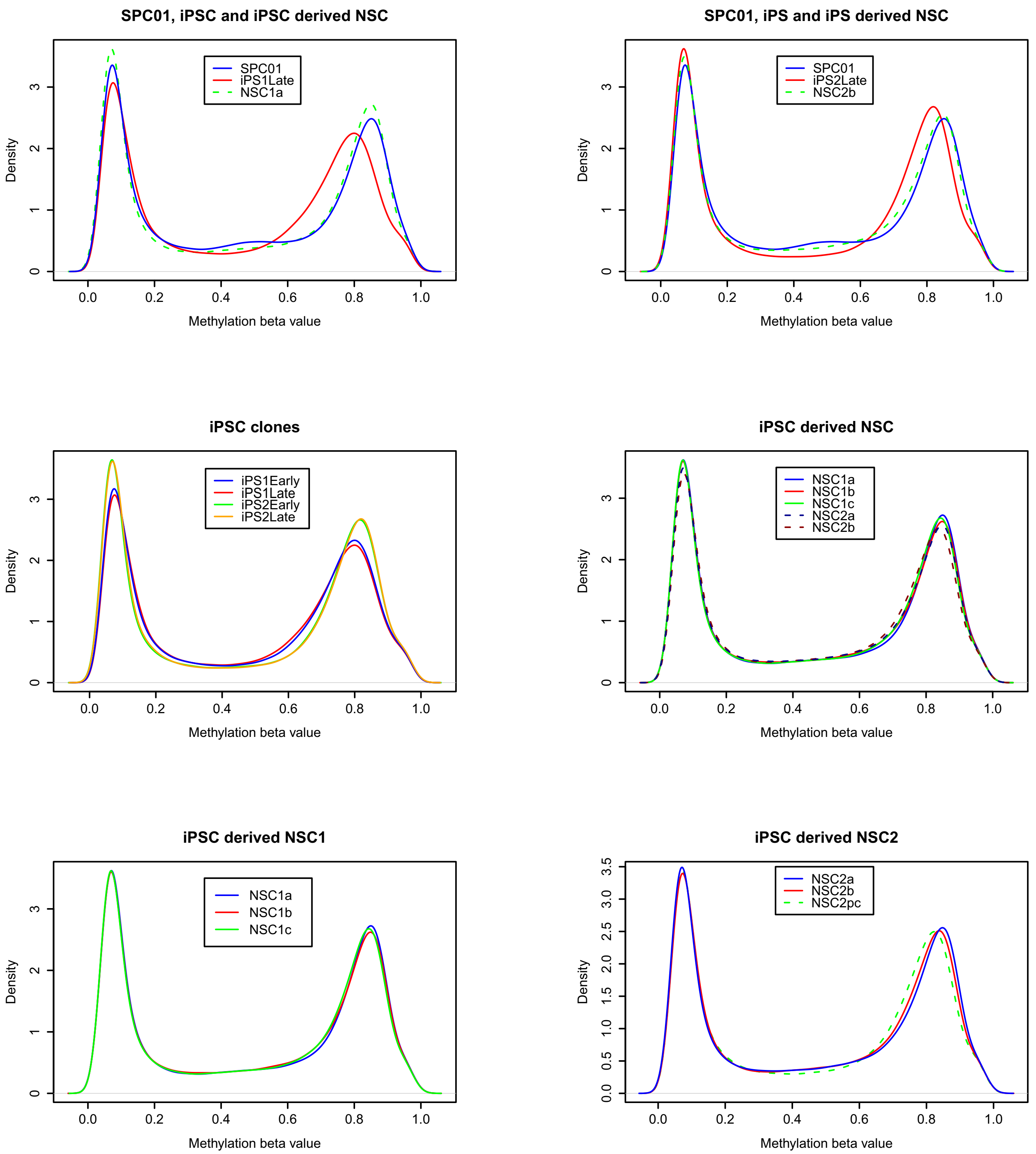

Figure S5. Autosomal CpG methylation beta value distributions

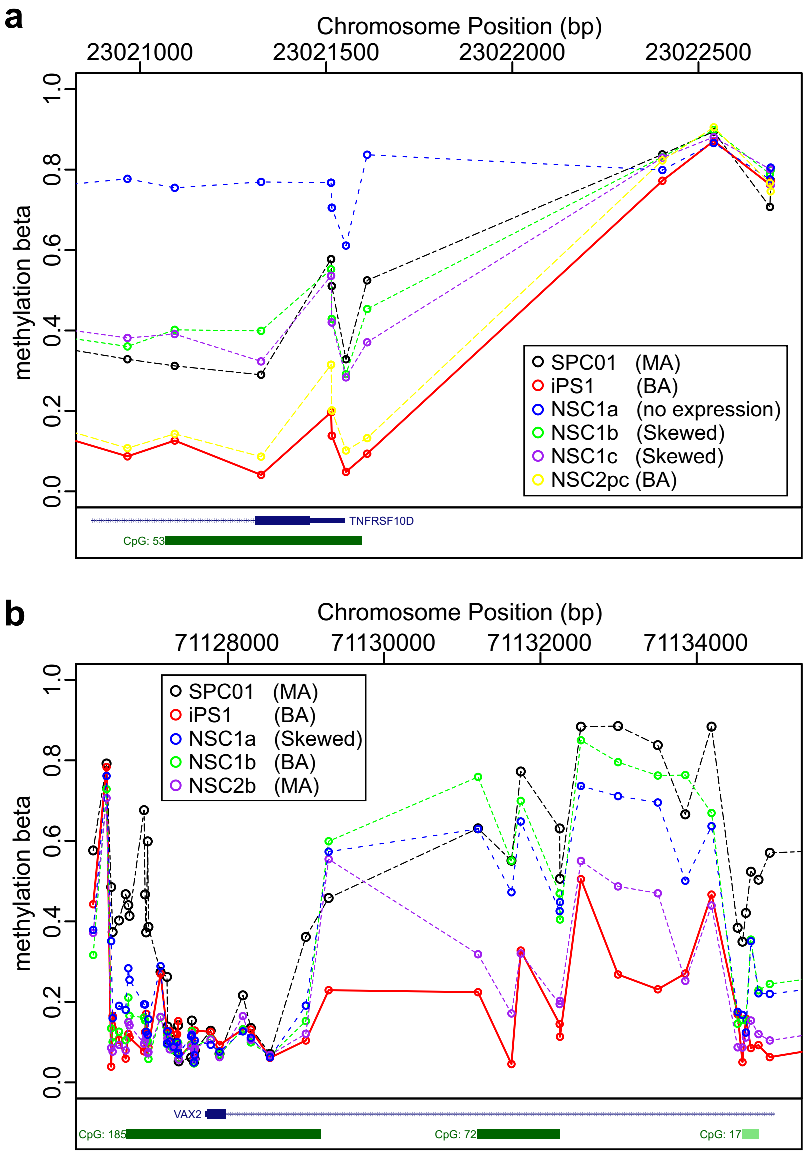

Figure S6 - Differential DNA methylation is seen across clones exhibiting different allelic expression patterns. The effect on DNA methylation sites for the SPC01 neural stem cell before and after reprogramming, together with iPS derived NSCs are shown in regions across the promoter, first exon and intronic region of (a) TNFRSF10D and (b) VAX2. The corresponding allelic expression status is indicated. Exons are represented by the blue boxes and introns by the arrowed lines. CpG islands are shown as a green box.

a

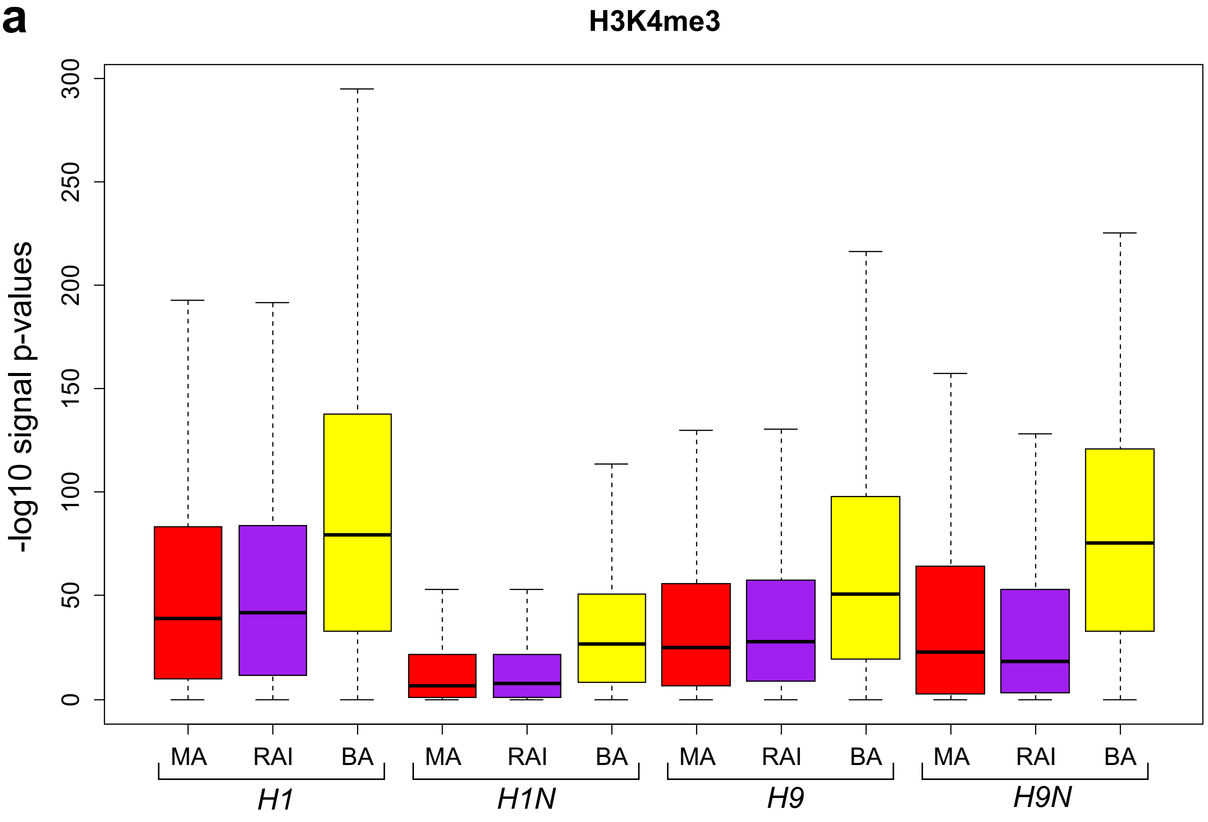

b

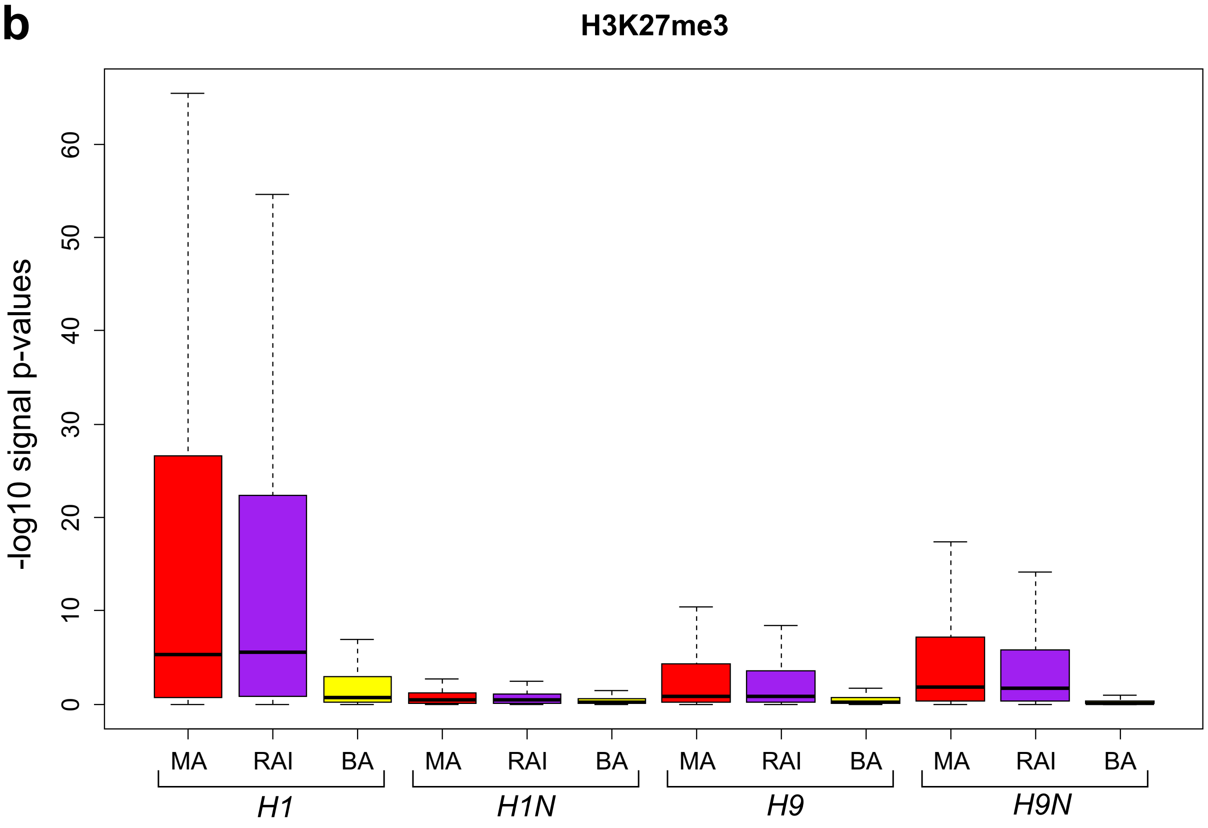

Supplementary Figure S7 - Promoter level chromatin status of ES cells and ES derived neural progenitor cells for gene loci identified in this study with differing allelic expression status. Full legend on the following page.

c

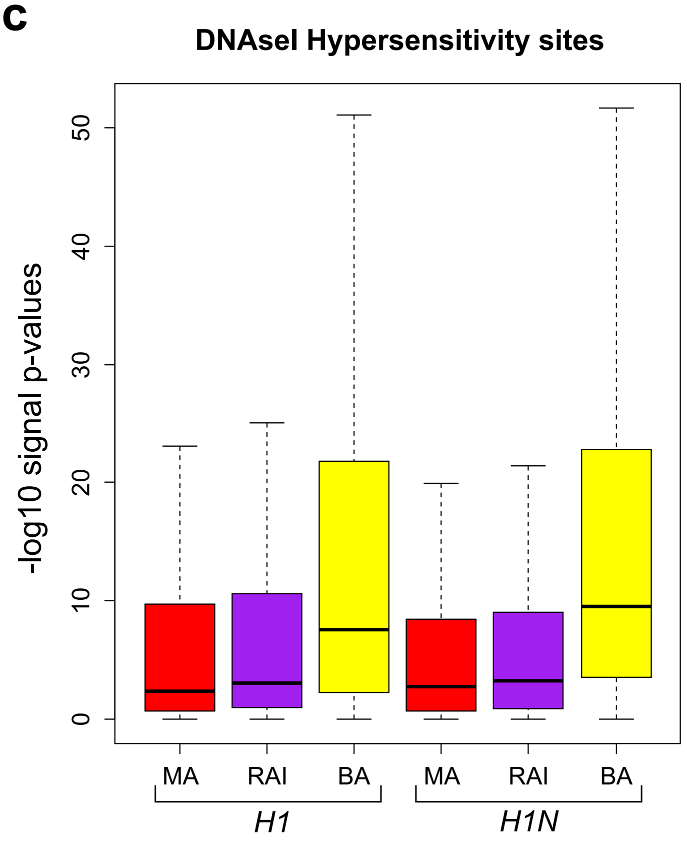

Supplementary Figure S7 - Promoter level chromatin status of ES cells and ES derived neural progenitor cells for gene loci showing monoallelic (MA), random allelic imbalance (RAI) and randomly chosen biallelic genes (BA) originally detected in iPS derived neural stem cells from this study (cells NSC1a, NSC1b, NSC1c, NSC2a, NSC2b). Chromatin data was sourced from the NIH Epigenomics Roadmap Mapping Consortium release 9 (http://egg2.wustl.edu/roadmap/web_portal/) and are show as -log10 p-value signal values from the promoter region (1000bp upstream and 500bp downstream of transcriptional start site). (a) shows the open chromatin mark H3K4me3 (b) shows the repressive chromatin mark H3K27me3 and (c) shows DNAse I hypersensitivity sites. Data were obtained for ES cells H1 and H9 as well as their H1 and H9 derived neural progenitor cells (H1N and H9N).

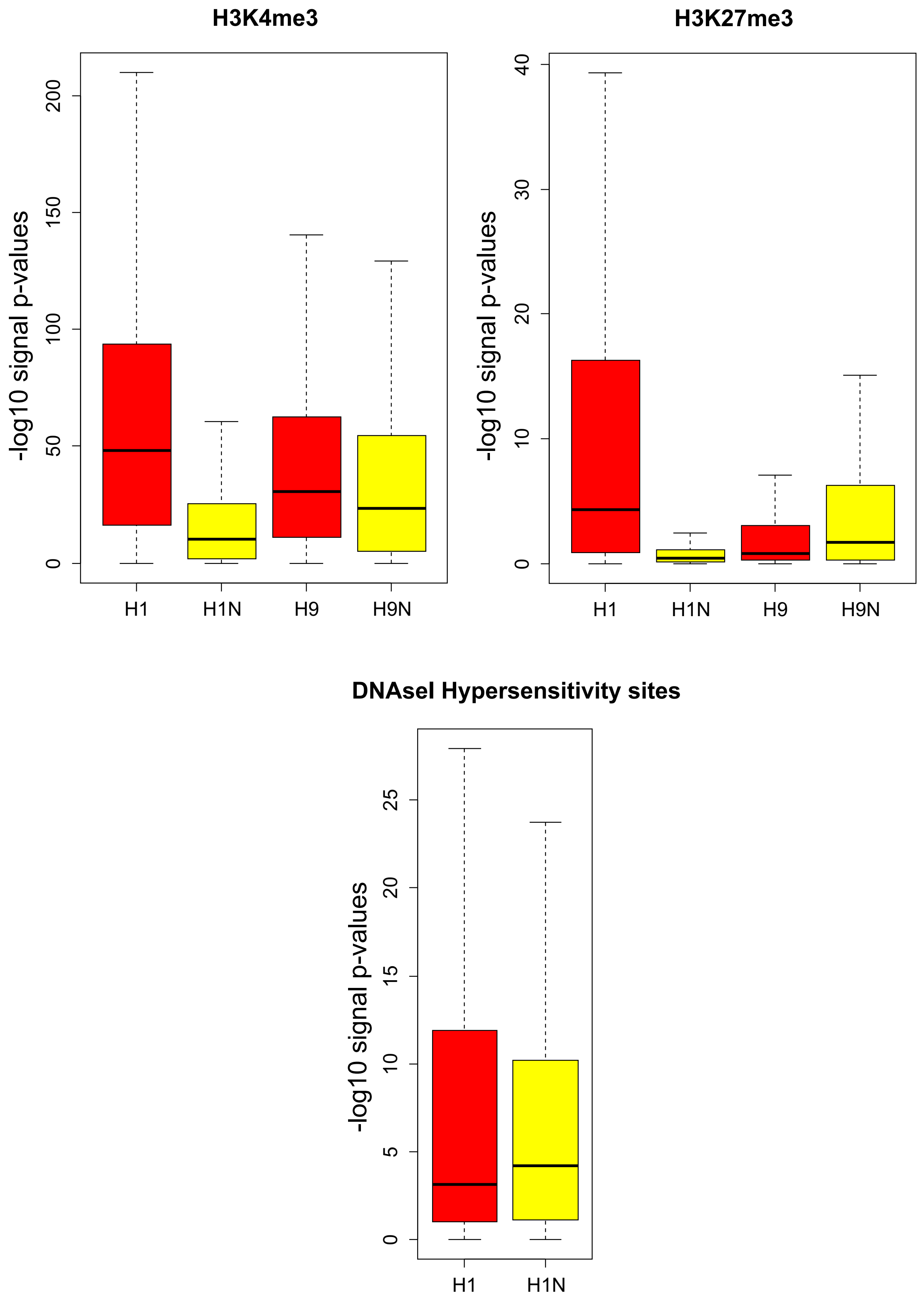

Supplementary Figure S8 - Promoter level chromatin status of ES cells and ES derived neural progenitor cells for gene loci showing biallelic expression in iPSC cells yet monoallelic expressing iPS derived neural stem cells. Chromatin data was sourced from the NIH Epigenomics Roadmap Mapping Consortium release 9 (http://egg2.wustl.edu/roadmap/web_portal/) and are show as -log10 p-value signal values. Data were obtained for ES cells H1 and H9 as well as their H1 and H9 derived neural progenitor cells (H1N and H9N).

[illegible]	**Wilcoxon Rank Sum p-value**	**n (MA,BA)**
TSS1500	0.528	88,116
TSS200	0.00601	75,99
5'UTR	0.08475	130,169
1stExon	$3.726x10^{-7}$	60,77
Body	0.003458	190,216
3'UTR	0.1547	19,27
CpG Island N_Shelf	1	13,11
CpG Island N_Shore	0.06444	55,76
CpG Island	$4.799x10^{-10}$	134,172
CpG Island S_Shore	0.7893	55,78
CpG Island S_Shelf	0.1683	12,13

Table S2. Wilcoxon Rank Sum Test on random monoallelic expressed genes from NSC1 monoallelic expressing clones versus biallelic expressing clones. The position of the probes in relation to the gene and CpG Island are shown together with the Wilcoxon rank sum p-value and the number of individual CpG probes included in each statistical test.

Analyzed Files

File Name	Pluri Raw	Pluri Logit P	Novelty	Novelty Logit P	PluriTest Result
iPSC1.Early	21.015	1	1.388	0.008	Pass
iPSC1.Late	23.465	1	1.38	0.008	Pass
iPSC2.Early	20.673	1	1.393	0.009	Pass
iPSC2.Late	24.584	1	1.48	0.021	Pass

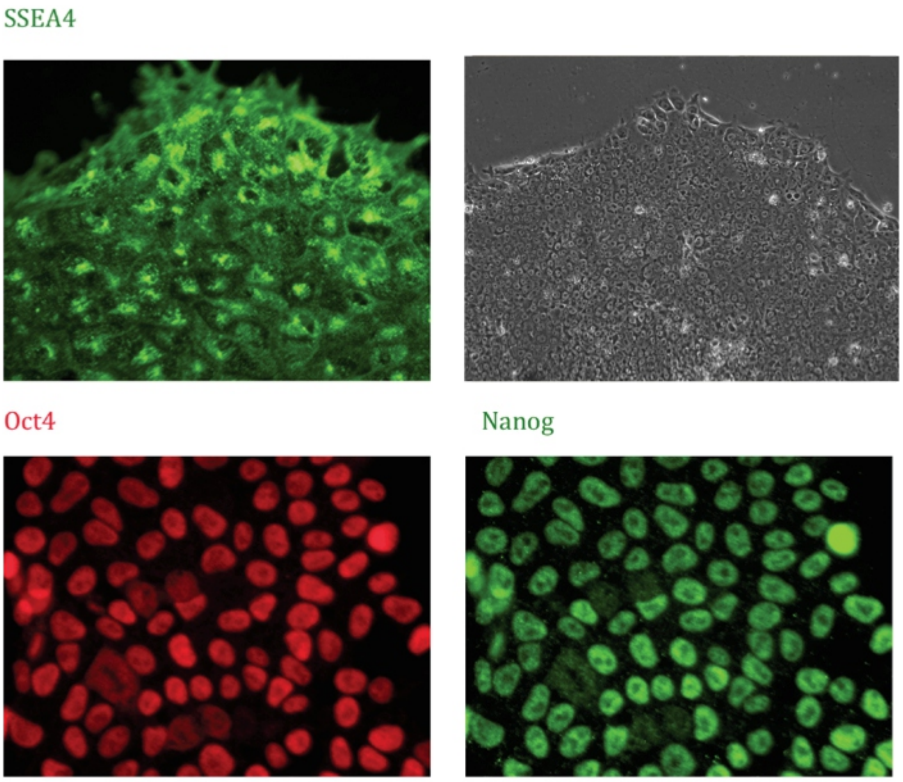

Figure S9. Pluripotency checks made by (a) gene expression analysis using the Pluritest (http://www.pluritest.org) and (b) immunohistochemistry staining of pluripotency markers.

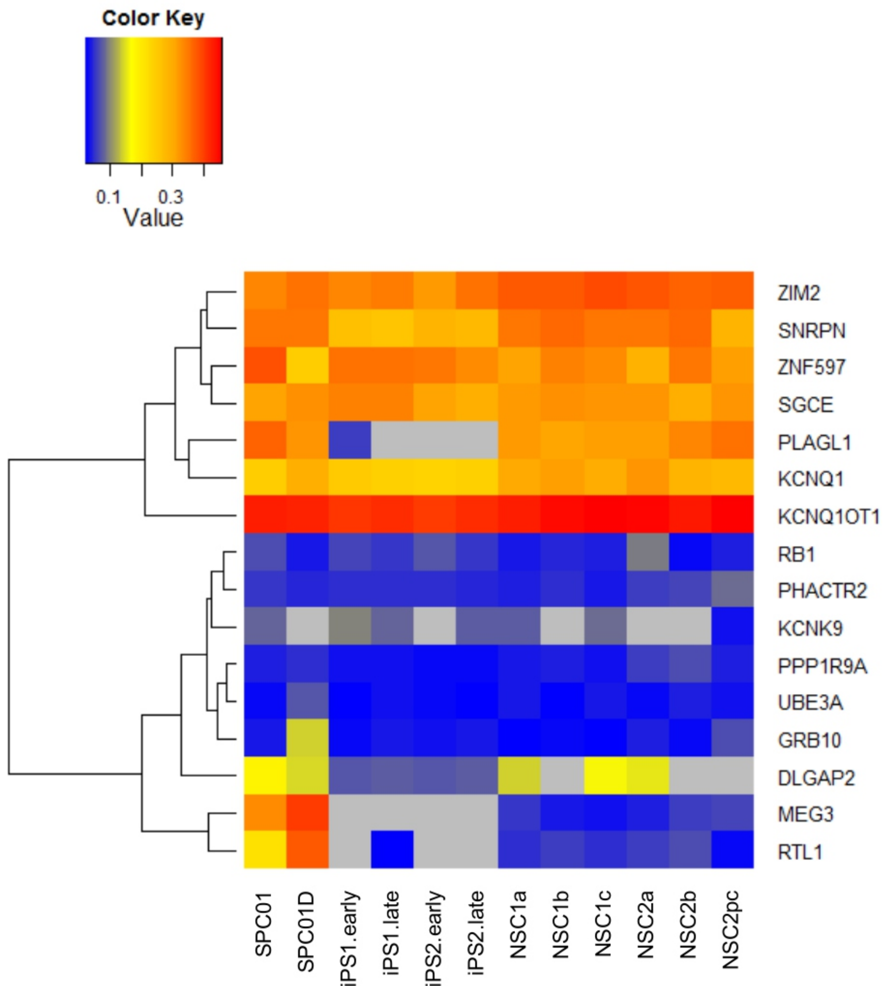

Figure S10. Allelic expression status of known imprinted genes from the Catalogue of Imprinted genes. Genes which originally showed monoallelic expression generally maintain this status when expressed, apart from MEG3 and RTL1 which show biallelic expression after epigenetic reprogramming and subsequent neuralisation. MEG3 and RTL1 belong to the DLK1 locus, a region previously reported as showing abberant imprinting regulation in iPSC cells (Stadfield et al, Nature. 2010 May 13;465(7295):175-81).

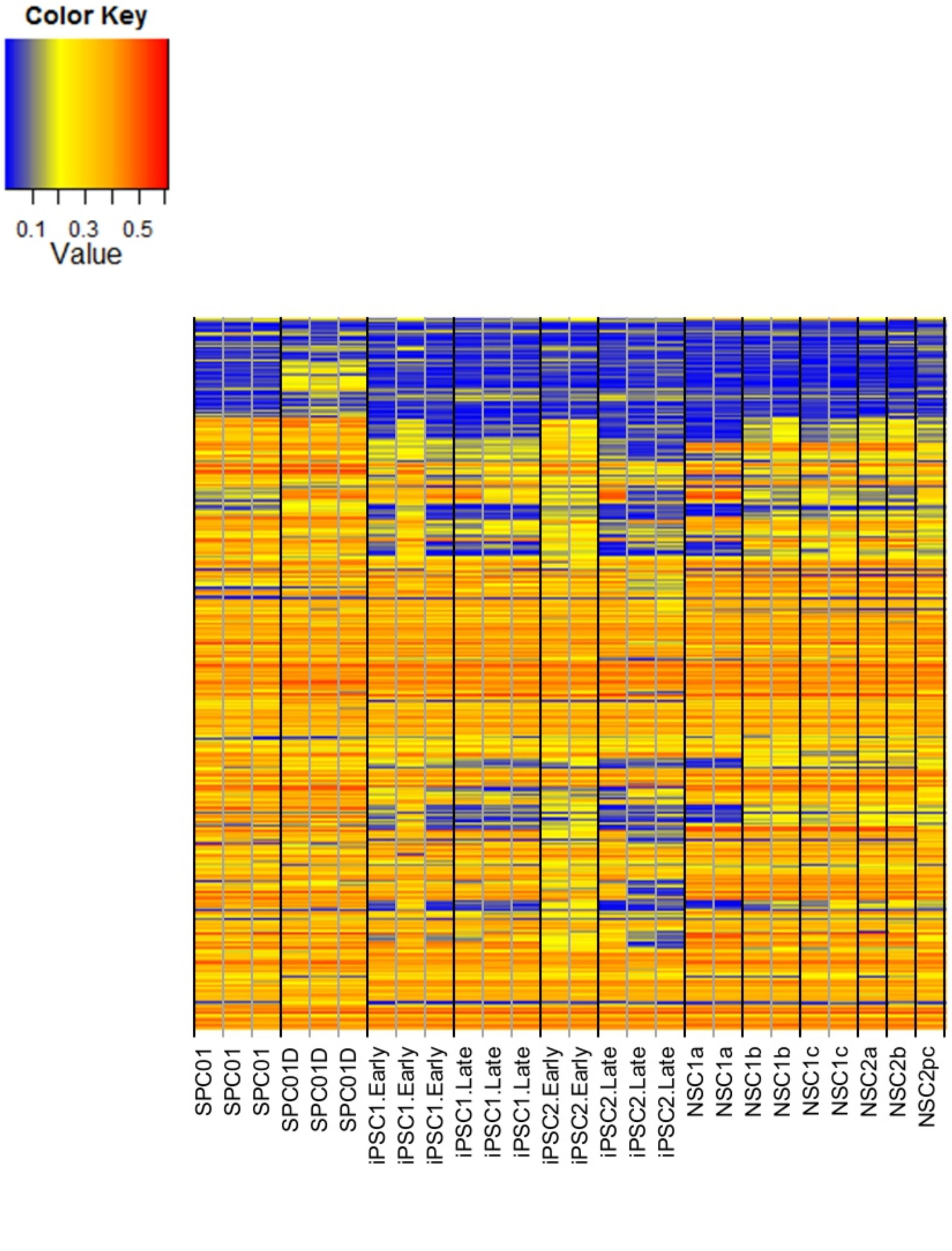

Figure S11. (a) Heatmap of allelic expression across the length of chromosome X derived from 930 SNP probes which showed detectable expression in all tested cell line. Yellow and red tones indicate monoallelic expression whereas blue represents biallelic expression. Profiling shows X inactivation is largely unchanged although some discrete regions show evidence of escaping X-inactivation in a few iPS clones and one NSC clone. This has previously been documented as an issue in iPSC cells (Mekhoubad et al., 2012, Cell Stem Cell 10, 595-609).
